# Integrative transcriptome analysis identifies deregulated microRNA-transcription factor networks in lung adenocarcinoma

**DOI:** 10.18632/oncotarget.8713

**Published:** 2016-04-12

**Authors:** Naiara C. Cinegaglia, Sonia Cristina S. Andrade, Tomas Tokar, Maísa Pinheiro, Fábio E. Severino, Rogério A. Oliveira, Erica N. Hasimoto, Daniele C. Cataneo, Antônio J.M. Cataneo, Júlio Defaveri, Cristiano P. Souza, Márcia M.C. Marques, Robson F. Carvalho, Luiz L. Coutinho, Jefferson L. Gross, Silvia R. Rogatto, Wan L. Lam, Igor Jurisica, Patricia P. Reis

**Affiliations:** ^1^ Department of Surgery and Orthopedics, São Paulo State University (UNESP), Botucatu, SP, Brazil; ^2^ Department of Animal Biotechnology, University of São Paulo (USP), Piracicaba, SP, Brazil; ^3^ Institute of Biosciences, University of São Paulo (USP), São Paulo, SP, Brazil; ^4^ Department of Genetics, São Paulo State University (UNESP), Botucatu, SP, Brazil; ^5^ Department of Biostatistics, São Paulo State University (UNESP), Botucatu, SP, Brazil; ^6^ Department of Pathology, São Paulo State University (UNESP), Botucatu, SP, Brazil; ^7^ Molecular Oncology Research Center, Barretos Cancer Hospital, Barretos, SP, Brazil; ^8^ Barretos School of Health Sciences, Barretos, SP, Brazil; ^9^ Department of Morphology, São Paulo State University (UNESP), Botucatu, SP, Brazil; ^10^ International Center of Research and Training (CIPE), A. C. Camargo Cancer Center, São Paulo, SP, Brazil; ^11^ Department of Urology, São Paulo State University, UNESP, Botucatu, SP, Brazil; ^12^ Department of Integrative Oncology, British Columbia Cancer Research Centre, Vancouver, BC, Canada; ^13^ Princess Margaret Cancer Centre, University Health Network, Toronto, ON, Canada; ^14^ Departments of Medical Biophysics and Computer Science, University of Toronto, Toronto, ON, Canada; ^15^ Experimental Research Unity (UNIPEX), Faculty of Medicine, São Paulo State University (UNESP), Botucatu, SP, Brazil

**Keywords:** lung adenocarcinoma, transcriptome sequencing, microRNAs, transcription factor networks, molecular targets

## Abstract

Herein, we aimed at identifying global transcriptome microRNA (miRNA) changes and miRNA target genes in lung adenocarcinoma. Samples were selected as training (*N* = 24) and independent validation (*N* = 34) sets. Tissues were microdissected to obtain >90% tumor or normal lung cells, subjected to miRNA transcriptome sequencing and TaqMan quantitative PCR validation. We further integrated our data with published miRNA and mRNA expression datasets across 1,491 lung adenocarcinoma and 455 normal lung samples. We identified known and novel, significantly over- and under-expressed (*p* ≤ 0.01 and FDR≤0.1) miRNAs in lung adenocarcinoma compared to normal lung tissue: let-7a, miR-10a, miR-15b, miR-23b, miR-26a, miR-26b, miR-29a, miR-30e, miR-99a, miR-146b, miR-181b, miR-181c, miR-421, miR-181a, miR-574 and miR-1247. Validated miRNAs included let-7a-2, let-7a-3, miR-15b, miR-21, miR-155 and miR-200b; higher levels of miR-21 expression were associated with lower patient survival (*p = 0.042*). We identified a regulatory network including miR-15b and miR-155, and transcription factors with prognostic value in lung cancer. Our findings may contribute to the development of treatment strategies in lung adenocarcinoma.

## INTRODUCTION

Global incidence data for cancers of the lung, bronchus and trachea estimates the occurrence of >1.8 million new cases with >1.5 million deaths every year, worldwide [1]. In the United States, 2012 incidence data for non-small cell cancers of the lung and bronchus estimated the occurrence of 41.48/100,000 new cases with an annual death rate of 44.96/100,000 individuals. The 5-year relative survival was ~22% from 2005-2011, indicating that lung cancer remains as a leading cause of cancer death [2]. A new histopathological classification of lung cancer has been established, as treatment strategies for patients with advanced disease should rely on histology and tumor molecular genotyping. Among the two major histological types, Non-Small Cell Lung Cancer (NSCLC) comprise the majority (~85%) of cases and is divided into histological subtypes, the most common being adenocarcinoma. Invasive lung adenocarcinoma is further classified by histological subtyping analysis, to determine its predominant histological pattern of lepidic, acinar, papillary, micropapillary or solid; a micropapillary pattern has been associated with poor prognosis [3].

Advances in the treatment of patients with lung adenocarcinoma were made with the introduction of molecularly targeted approaches, such as the use of tyrosine-kinase inhibitors for patients with tumors containing activating, sensitizing *EGFR* mutations [4] and Crizotinib for *ALK* rearrangements [5]. Recent data from The Cancer Genome Atlas (TCGA) [6] and The Lung Cancer Mutation Consortium (LCMC) [7] demonstrated the importance of tumor genotyping in therapeutic decision for patients with lung adenocarcinoma. LCMC data showed that actionable mutations in genes such as *EGFR*, *K-RAS*, *N-RAS*, *ALK*, *ERBB2*, *BRAF*, *PIK3CA*, *AKT, MEK1* and *MET* amplification, were found in >60% of lung adenocarcinomas and patients who received treatment guided by tumor genotyping lived longer compared to patients who did not receive targeted treatment [7]. Moreover, candidate driver mutations were found in *TP53*, *KEAP1*, *NF1* and *RIT1* in tumors lacking oncogene mutations [6]. Identification of genetic drivers is thus essential to establish efficient tumor genotyping at the diagnostic level, in order to tailor patient treatment.

Although several studies have identified driver mutations with a therapeutic role in lung adenocarcinoma, ~40% of such changes are yet unidentified [8–10]. As molecularly targeted approaches have benefited a fraction of patients with specific tumor histology classification and genetics, the need remains to identify new targets for further improving treatment decisions.

miRNAs are small, non-coding RNAs (~18-22 nucleotides) transcribed from DNA and with a role in gene expression regulation mainly leading to translational repression [11]. miRNAs play roles in multiple biological processes, such as embryonic development, cell proliferation and differentiation [11] and tumorigenesis, acting as oncogenes and tumor suppressor genes [12]. Deregulated miRNA expression has been associated with lung tumorigenesis [13–15]. To the best of our knowledge, ours is the first study on global transcriptome miRNA sequencing of lung adenocarcinoma from Brazilian patients, extending to validation in an independent sample set as well as across multiple high-throughput miRNA and gene expression datasets. By using stringent criteria on sample selection and data analysis, we were able to identify novel miRNAs that are deregulated exclusively in tumors and thus related to tumorigenesis.

## RESULTS

### Deregulated expression of miRNAs identified by miRNA-sequencing (miRNA-Seq)

miRNA-Seq generated 13,135,522 reads with an average of 547,313 reads/sample. FastQC quality test showed that 96.1% (12,623,236.642) of reads had a Q-score ≥30 and were thus considered for further analyses. Overall unpaired sample analysis showed that 11 miRNAs were statistically significantly (*p* ≤ 0.01 and FDR ≤ 0.1) deregulated, including miR-486 under-expression and let-7a-2, let-7g, miR-15b, miR-181b-1, miR-181b-2, miR-23b, miR-26a-1, miR-26a-2, miR-26b and miR-93 over-expression in the tumor compared to normal tissues. In addition, a paired-sample analysis (tumor and normal from same patients) showed that 22 miRNAs were statistically significantly deregulated (*p* ≤ 0.01; FDR ≤ 0.1); 8 miRNAs (miR-486, miR-1247, miR-218-1, miR-181a-1, miR-181a-2, miR-328, miR-574 and miR-886) were down-regulated and 14 miRNAs (let-7a-3, miR-146b, miR-26a-1, miR-200b, miR-191, miR-181c, miR-10a, miR-155, miR-99a, miR-30e, miR-21, miR-425, miR-29a and miR-421) were up-regulated in the tumor compared to the normal tissue from the same patient. Notably, deregulated expression of miR-486 and miR-26a-1 were detected in both analyses (unpaired and paired samples), considering filtering criteria of *p* ≤ 0.01 and FDR ≤ 0.1. Statistically significantly deregulated miRNAs identified in unpaired and paired samples are shown in Table 1.

**Figure 1:**
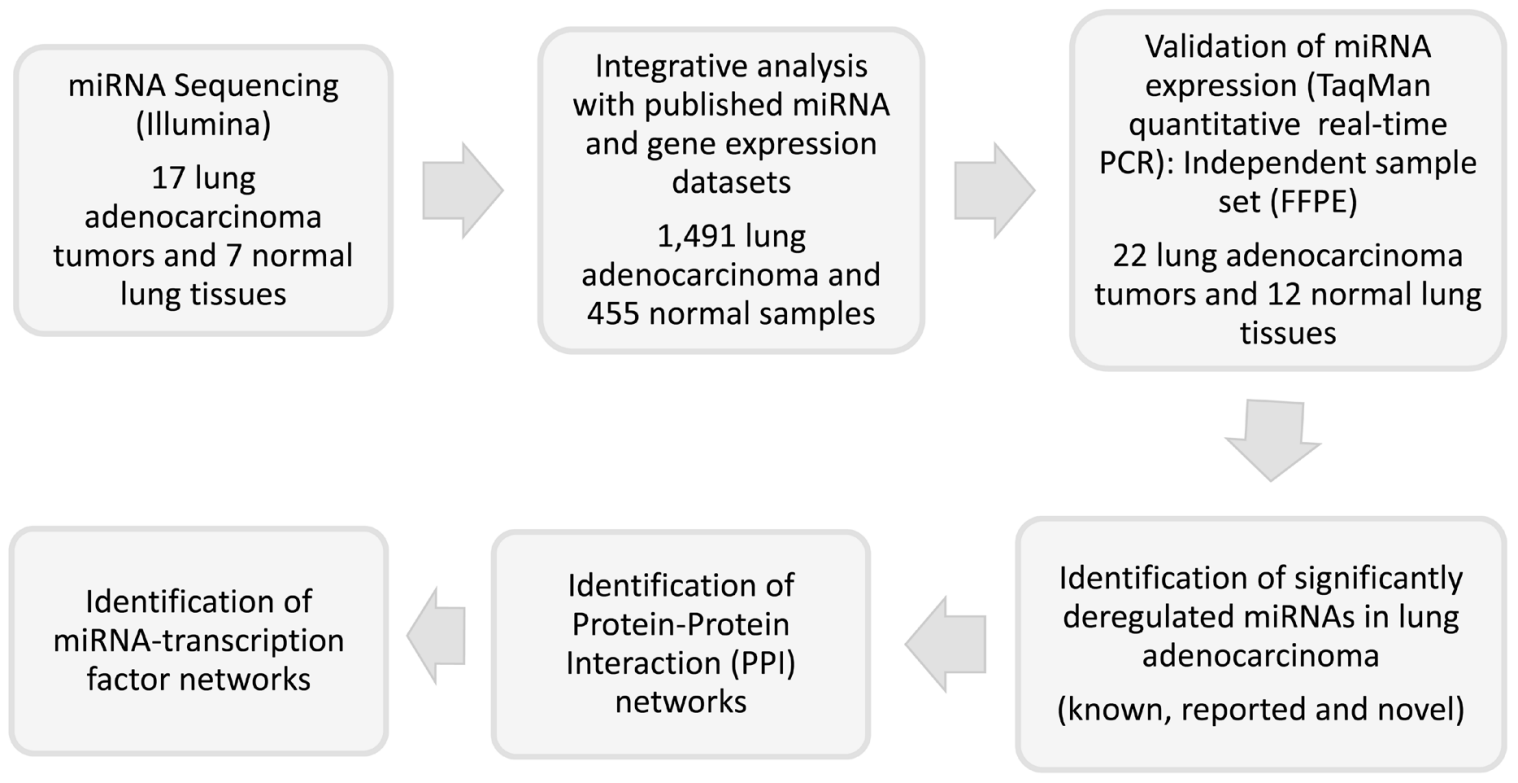
Experimental design

Experimental design and data analysis steps are outlined in Figure 1.

**Table 1:** Deregulated miRNAs in lung adenocarcinoma compared to normal lung tissues in unpaired and paired sample analysis

miRNA	LogFC	*P*-value	FDR
**Unpaired samples**			
miR-486	−1.635	0.0004	0.0689
let-7g	0.8879	0.0015	0.0689
miR-15b	0.9831	0.0004	0.0689
miR-181b-2	0.9887	0.0009	0.0689
miR-23b	0.9479	0.0014	0.0689
miR-26a-1	0.9756	0.0014	0.0689
miR-26a-2	0.9595	0.0013	0.0689
miR-26b	1.0565	0.0009	0.0689
miR-93	1.203	0.0005	0.0689
let-7a-2	0.8844	0.0025	0.1052
miR-181b-1	0.9221	0.0027	0.1052
**Paired samples**			
miR-486	−2.5430	8.8276E-12	1.805E-09
miR-1247	−2.6582	0.0003	0.0033
miR-218-1	−1.6175	0.0001	0.0043
miR-181a-2	−1.1145	0.0002	0.0079
miR-328	−1.4708	0.0004	0.0108
miR-181a-1	−1.1444	0.0006	0.0147
miR-574	−1.1719	0.0036	0.0503
miR-886	−1.3187	0.0046	0.0575
let-7a-3	0.4856	3.2638E-264	5.1692E-262
miR-146b	0.7012	0.0005	0.0147
miR-26a-1	0.7382	0.0043	0.0551
miR-200b	1.0898	0.0072	0.0864
miR-191	1.0935	0.0018	0.0307
miR-181c	1.1230	0.0041	0.0551
miR-10a	1.2858	0.0009	0.0208
miR-155	1.3089	0.0029	0.0418
miR-99a	1.4447	0.0028	0.0418
miR-30e	1.4926	0.00002	0.0011
miR-21	1.5219	0.0028	0.0418
miR-425	1.6206	0.0076	0.0005
miR-29a	1.6488	0.0001	0.0043
miR-421	1.7648	0.0085	0.0993

LogFC = log (base 2)fold change; FDR = false discovery rate. FDR ≤ 0.1, *p* ≤0.01.

### Integrative analyses of our data with published datasets

Differentially expressed miRNAs identified herein were integrated with previously published datasets. We found deregulated miRNAs that were also consistently reported by previous studies (we refer to these as “known”): 2 over-expressed: miR-21, miR-191 and 2 under-expressed: miR-218 and miR-486. Additionally, we identified deregulated miRNAs that were previously reported by at least one study (we refer to these as “reported”): 5 over-expressed: let-7g, miR-93, miR-155, miR-200b and miR-425, and 1 under-expressed: miR-328. The remaining deregulated miRNAs we identified have not been previously reported in lung adenocarcinoma compared to normal lung tissue (we refer to these as “novel”): 13 over-expressed: let-7a, miR-10a, miR-15b, miR-23b, miR-26a, miR-26b, miR-29a, miR-30e, miR-99a, miR-146b, miR-181b, miR-181c, miR-421 and 3 under-expressed: miR-181a, miR-574 and miR-1247. Statistical significance of overlap between our findings and consistently appearing miRNA reports was evaluated by hypergeometric test, resulting in *p* = 7.46E-5 and *p* = 1.48E-6 for over- and under-expressed miRNAs, respectively.

### miRNA-gene targets network

Next, we analyzed deregulated miRNAs and validated consistency of differential expression of their targets. Comparison of our data with multiple publicly available gene expression datasets allowed us to identify consistently deregulated genes in lung adenocarcinoma compared to normal lung tissues. We then assembled an interaction network between deregulated miRNAs and their target genes, including transcription factors participating on these interactions. We then analyzed statistical significance of enrichment of the downstream/upstream neighborhoods of order 2 (n^2^_down_/n^2^_up_) of deregulated miRNAs/genes by deregulated genes/miRNAs. Since miRNAs act mainly as inhibitors, for down-regulated miRNAs only up-regulated genes were taken into consideration and for up-regulated miRNAs only down-regulated genes were considered.

Recently, it has been shown that biological pathways, or Gene Ontology (GO) terms may be falsely identified as significantly enriched even by target genes of randomly selected miRNAs [16, 17]. This effect originates from knowledge bias, due to which miRNA-target pairs are discovered (e.g. computationally predicted) with a higher rate if the miRNA or target gene is known to be associated with specific biological processes (e.g. cell cycle) or diseases (e.g. cancer). Therefore, the rates of false positive and false negative (missing) miRNA-target predictions are not distributed equally among the genes/miRNAs and depend on their biological properties, leading to accumulation of false predictions within certain biological contexts [16]. We assume that this problem is not affecting the results of the two enrichment analyses described above, since rates of false predictions are presumably equal among the deregulated *vs*. non-deregulated miRNAs/target genes, which were identified experimentally and validated in independent sample cohorts.

We identified 11 miRNAs whose n^2^_down_ is significantly enriched (*p < 0.05*) by deregulated genes; deregulation of these miRNAs may play an important role in lung adenocarcinoma, leading to gene expression changes. Some of these miRNAs are consistently reported (miR-21, miR-191), or have been reported at least once (miR-200b, miR-93). The remaining miRNAs: miR-15b, miR-23b, miR-29a, miR-30e, miR-146b, miR-181c are novel. We have applied two thresholds for statistical significance in order to identify genes whose upstream neighborhood was significantly enriched by deregulated miRNAs. Interestingly, we identified 705 genes whose n^2^_up_ was significantly enriched (*p < 0.05*) by deregulated miRNAs. Out of these 705 genes, we identified 148 genes whose n^2^_up_ was significantly enriched (*p* < 0.001), involving at least two deregulated miRNAs. It is therefore reasonable to assume that deregulation of these 148 genes may be due to the differential expression of their regulating miRNAs. The list of 148 genes is provided in Supplementary Table S1. Next, in order to construct the physical protein-protein interaction PPI network (as described below), we enforced the presence of at least two deregulated miRNAs in the upstream neighborhood of each of the 148 genes.

### Protein-protein interaction networks in lung adenocarcinoma, linking deregulated miRNA target genes

Using the 148 genes (miRNA-deregulated targets; Supplementary Table S1) we constructed the corresponding physical protein-protein network (see Methods). Resulting PPI network (Supplementary Figure S1) comprised 4,324 nodes, among which 469 (*p = 0.036*, random network generation) comprise a list of “prognostic genes”, which are genes derived from lung prognostic signatures, downloaded from Cancer Data Integration Portal (CDIP) database (http://ophid.utoronto.ca/cdip), and used earlier in [12]. We found that 58/148 genes were directly connected by PPIs (*p = 0.044*, random network generation), showing that miRNA-deregulated targets are tightly connected on the PPI level. These miRNAs are highly interconnected in the PPI networks, coordinating the expression of several different proteins (Supplementary Figure S2).

### Validation of deregulated expression of novel and known miRNAs in lung adenocarcinoma

TaqMan PCR validation was performed for all significantly deregulated miRNAs in an independent set of 22 lung adenocarcinoma samples and 12 normal lung tissues. Fourteen miRNAs showed concordant levels of expression between miRNA-Seq and TaqMan PCR data (Table 2). 5/14 miRNAs were under-expressed (miR-486, miR-181a-1, miR-181a-2, miR-218-1 e miR-886) and 9 were over-expressed (let-7a-2, let-7a-3, let-7g, miR-15b, miR-26b, miR-200b, miR-155, miR-21, miR-425). 6/14 miRNAs; let-7a-2, let-7a-3, miR-15b, miR-200b, miR-155 and miR-21 were statistically significantly deregulated (*p* ≤ 0.01; FDR ≤ 0.1) in tumor compared to normal in both miRNA-Seq and TaqMan PCR analyses (Table 2).

**Table 2:** Deregulated miRNAs in both miRNA-Seq and TaqMan PCR analyses

miRNA	miRNA-Seq logFC	*P-value*	miRNA	TLDA logFC	*P-value*
let-7a-2	0.8844	0.0025	let-7a	2.3601	0.000*
let-7a-3	0.4856	3.2638E-264	let-7a	2.3601	0.000*
miR-15b	0.9831	0.0004	miR-15b	2.4022	0.000*
miR-200b	1.0898	0.0072	miR-200b	1.9404	0.000*
miR-21	1.5219	0.0028	miR-21	2.6311	0.001*
miR-155	1.3089	0.0029	miR-155	1.3294	0.010*
miR-486	−1.635	0.0004	miR-486	−3.9434	0.084
miR-181a-1	−1.1444	0.0006	miR-181a	−0.8625	0.140
miR-181a-2	−1.1145	0.0002	miR-181a	−0.8625	0.140
let-7g	0.8879	0.0015	let-7g	0.3918	0.143
miR-26b	1.0565	0.0009	miR-26b	0.5597	0.224
miR-218-1	−1.6175	0.0001	miR-218	−1.4699	0.386
miR-886	−1.3187	0.0046	miR-886-3p	−1.1746	0.482
miR-425	1.6206	0.0076	miR-425-5p	0.1190	0.906

Test sample set: *p* ≤ 0.01 and FDR ≤ 0.1, as determined by EdgeR software.

Validation sample set: *p* ≤ 0.01 as determined by Expression Suite software.

* Statistically significantly deregulated miRNAs identified in both test and validation sets by transcriptome sequencing and TaqMan PCR analyses.

**Figure 2:**
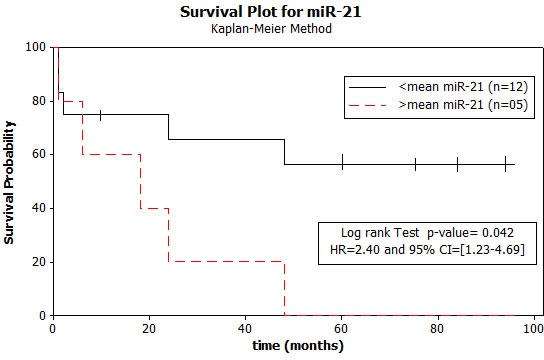
Kaplan-Meier survival analysis Patients (training set) with tumors showing higher than average miR-21 expression levels had significantly poorer survival compared to patients who are alive.

Statistically significant correlations were identified between female gender and over-expression of let-7a-2 (*p = 0.0062*), let-7a-3 (*p = 0.0052*) and miR-15b (*p = 0.0294*). Over-expression of let-7a-2, let-7a-3 and miR-15b was associated with poorly differentiated tumors (*p = 0.0245*). Interestingly, miR-21 levels were higher in tumors from patients who died of disease compared to patients who are alive with disease (*p = 0.042*) (Figure 2).

## DISCUSSION

Herein, by applying stringent criteria to our miRNA-Seq and TaqMan PCR analyses, we identified and validated deregulated expression of miRNAs let-7a-2, let-7a-3, miR-15b, miR-21, miR-155 and miR-200b in lung adenocarcinoma compared to histologically normal lung tissues. Integrative analyses of our results allowed us to identify consistently deregulated miRNAs in lung adenocarcinoma across different high-throughput published datasets. We identified deregulated miRNAs that have not been previously reported in lung adenocarcinoma. The identification of novel miRNAs was possible mainly due to the use of very stringent sample selection criteria, having at least 90% of tumor or normal cells in the tissues used for deep sequencing and validation analyses. Among the novel miRNAs, let-7a and miR-15b were identified and validated in an independent sample set. Deregulated miRNAs including novel, known and reported miRNAs (let-7a-2, let-7a-3, miR-15b, miR-21, miR-155 and miR-200b) act by silencing the expression of tumorigenesis-related genes.

Although validation data for other miRNAs was not statistically significant, both miRNA-Seq and TaqMan data showed concordant levels of miRNA expression in tumors compared to normal tissues. This lack of statistical significance may be due to differences in sample sources (fresh-frozen *vs*. formalin-fixed, paraffin embedded FFPE tissues) used for sequencing and validation analyses, respectively. FFPE tissues represent a valuable resource for cancer studies, as these samples can provide long-term patient follow-up, including information on treatment response and survival. Although formalin fixation causes nucleic acid degradation and cross linking of proteins to DNA, several studies reported useful and reproducible molecular genetic data using FFPE compared to frozen samples [18, 19]. This is likely due to improved RNA extraction protocols designed for FFPE tissues. Additionally, as miRNAs are small molecules and protected by the RISC complex, they are less susceptible to degradation [20, 21]. A previous study showed that the TaqMan Human MicroRNA Array platform is suitable for analysis of FFPE tissues with high reproducibility (*r* = 0.95 between duplicates, *p < 1e-5*) [22]. Therefore, proper use of both frozen and FFPE tissues and controls is an important sample resource to improve statistical power in discovery and validation studies.

Our results showed increased levels of let-7a variants (let-7a2 and let-7a3) in lung adenocarcinoma. As let-7 family of miRNAs (let-7a, b, c, d, e, f, g and i) has been reported as under-expressed and suggested to repress cancer cell growth and proliferation, including lung squamous cell carcinoma [23–25], it remains to be investigated whether increased expression levels of let-7 family could have a role in lung adenocarcinoma. Landi *et al*. [26] showed higher levels of let-7 family members in adenocarcinoma compared to squamous cell carcinoma subtype. Considering let-7 tumor suppressive functions, let-7 family members may influence mostly lung squamous cell carcinoma than adenocarcinoma. Additionally, differential expression levels of let-7 family distinguished lung adenocarcinoma from squamous cell carcinoma [26]. Our data confirmed that increased let-7a levels may be specific to the adenocarcinoma subtype.

miR-15b over-expression was detected in the blood of patients with NSCLC and deregulated expression of miR-15b and miR-27b, combined, was able to distinguish patients with NSCLC from healthy individuals [27]. Novel miRNAs identified herein, including let-7a and miR-15b were correlated with lung adenocarcinoma compared with lung squamous cell carcinoma. Interestingly, miR-15b/16-2 up-regulation was shown to activate genes involved in DNA repair pathways; *PPM1D* (*WIP1*; wild-type p53-induced phosphatase 1) was shown as a direct target of miR-15b, suggesting that DNA damage response by miR-15b may be partially modulated by *PPM1D* inhibition [28]. *PPM1D* encodes a serine/threonine phosphatase that plays a role in dephosphorylation of several DNA damage-response proteins such as ATM, ATR, *p38*MAPK, CHK1 and CHK2 [29]. MAPK and PI3K pathways activation was associated with known mutations in a small fraction of lung adenocarcinomas, suggesting other mechanisms of pathway activation during tumorigenesis [6], which could include post-transcriptional regulation by miRNAs.

Over-expression of miR-21 and mir-200b was detected in tumor and sputum of patients with early stage lung adenocarcinoma; a 4-miRNA signature (including miR-21 and miR-200b) distinguished patients with adenocarcinoma and squamous cell carcinoma from healthy individuals, with higher specificity and sensitivity for the adenocarcinoma subtype [30]. Interestingly, miR-21 over-expression and PTEN protein under-expression were associated with low sensibility to TKIs Gefitinib or Erlotinib and low survival of patients with NSCLC. Increased miR-21 and decreased PTEN expression was detected in Gefitinib-resistant cell lines with a reduced sensibility to Gefitinib due to PTEN inhibition and AKT/ERK activation while miR-21 inhibition was able to restore sensitivity to treatment [31]. We found that higher miR-21 levels were significantly associated with poorer patient survival. miR-21 has been identified as over-expressed in glioblastoma and to play a role in apoptosis, since suppression of miR-21 triggered activation of caspases 3 and 7 and increased programmed cell death in glioblastoma cells [32] thus demonstrating that miR-21 over-expression contributes to glioblastoma oncogenesis by silencing apoptosis-related genes.

miRNAs regulate pathways associated with disease progression and metastasis, such as TGF-β signaling, which activates transcription factors responsible for epithelial to mesenchymal transition (EMT). miR-200 family plays an important role in EMT through inhibition of *CDH1*, *ZEB1* and *ZEB2* (Zinc finger E-box binding homeobox) [33]. miR-200b over-expression inhibited the transcriptional repressor *ZEB2* and *CDH1* in breast carcinoma cells. *ZEB2* cooperates with TGF-β signaling and EMT through *CDH1* [34]. Although *ZEB1*/*CDH1* are repressed by miR-200b, restoration of *ZEB1* expression in breast cancer cells expressing miR-200b was unable to modify their metastatic potential, suggesting additional mechanisms underlying metastasis [35].

Wnt/β-catenin signaling has been associated with miR-155 in liposarcoma; CK1α (casein kinase 1α), a key regulator of Wnt/β-catenin pathway, is targeted by miR-155, leading to β-catenin signaling and *CCND1* activation, cell proliferation and liposarcoma progression [36]. miR-155 up-regulation has been reported in lung adenocarcinoma [37], detected in serum from patients with advanced-stage (IV) NSCLC and associated with low patient survival [38]. miR-155 may be a potential therapeutic target in cancer, as *in vitro* and *in vivo* data showed efficient delivery of anti-miR-155 in a hepatocellular carcinoma cell line [39]. miRNAs control gene expression either directly [40] or indirectly by targeting its upstream transcription factors. We showed a complex miRNA-transcription factor regulatory network composed, in part, of novel, differentially expressed miRNAs (miR-15b, miR-23b, miR-29a, miR-30e, miR-146b, miR-181b, and miR-181c).

We identified 705 genes which have been consistently reported as deregulated in lung adenocarcinoma and whose upstream neighborhood was significantly enriched by differentially expressed miRNAs. Of these 705 genes, 148 genes may be deregulated due to the differential expression of their regulatory miRNAs, since these genes passed stringent statistical data analysis criteria. Notably, 48 of these 148 genes are found in lung cancer prognostic signatures identified through the Cancer Data Integration Portal (CDIP) database. Among the 19 transcription factors identified herein (*EGR1*, *AP2C*, *FLI1*, *TAL1*, *GATA2*, *HMGA1*, *ERG*, *JUN*, *FOS*, *GCR*, *NFYA*, *TYY1*, *MEF2A*, *VDR*, *P63*, *JUND*, *NF2L2*, *HXA5* and *EPAS1*), *HMGA1* (high mobility group AT-hook 1) chromatin remodeling protein is highly expressed in poorly differentiated, aggressive tumors (reviewed in [41]), and has been identified as a lung cancer prognostic gene [42]. Increased *HMGA1* gene and protein expression was identified in NSCLC; HMGA1 protein over-expression was associated with disease stage, tumor grade, T category, nodal status and distant metastasis; patients with tumors over-expressing HMGA1 had lower survival [43] indicating that HMGA1 may have prognostic value in NSCLC.

miRNAs identified herein may be subjected to functional validation studies in order to assess their individual role in lung tumorigenesis. However, it is important to emphasize that functionality measures of individual miRNAs may be linked to the global functionality and coordinated actions of miRNA-regulated gene networks [44].

Our data corroborate known information on deregulated expression of miRNAs and identify novel deregulated miRNAs in lung adenocarcinoma. Novel miRNAs identified in tumors from Brazilian patients is a unique aspect of our study. Our data thus provide a distinctive and valuable contribution to the understanding of miRNA deregulation in lung adenocarcinoma. Our findings may lead to further clinical relevance by contributing to the development of novel therapeutic strategies for patients with lung adenocarcinoma.

## MATERIALS AND METHODS

### Ethics statement

This study was performed in accordance with the ethical standards and to the Declaration of Helsinki and according to national and international guidelines. Our study has been approved by the Research Ethics Boards of the Faculty of Medicine, UNESP, Botucatu, SP (4319/2012), AC Camargo Hospital, São Paulo, SP (1573/11) and Barretos Cancer Hospital, Barretos, SP (75907). Informed consent was obtained from all patients before sample collection.

### Patient samples

Inclusion criteria were patients >18 years old, histopathological diagnosis of lung adenocarcinoma, untreated before surgery. Exclusion criteria were patients < 18 years old and with diagnosis of other diseases. Samples were selected as training and validation sets. Training set samples (*N* = 24) were prospectively collected from surgeries performed at AC Camargo Hospital, SP (*N* = 17 lung adenocarcinoma samples and 7 histologically normal lung tissues from same patients). Prospectively collected samples were immediately frozen in liquid nitrogen and kept at −80°C until RNA extraction. Validation set samples (*N* = 34) were retrospectively obtained (2000-2012) from the Pathology Department, Faculty of Medicine, UNESP, Botucatu, SP and Barretos Cancer Hospital, Barretos, SP. Validation set samples comprised FFPE tissue blocks from lung adenocarcinoma (*N* = 22) and histologically normal lung tissues from same patients (*N* = 12). We aimed at identifying global miRNA expression changes in lung adenocarcinoma through transcriptome sequencing followed by TaqMan quantitative real-time PCR validation. Table 3 shows the detailed clinical and histopathological data of patients.

**Table 3:** Clinical and histopathological data of patients (training and validation sets)

Variables	Total Number (training)	*N* (%)	Total Number (validation)	*N* (%)	*p-value*
**Age (years)**					
Median (range)	66.8 (43-83)		58.0 (10-84)		
Mean	65		60.5		0.30
**Gender**					
Male	8	47	12	55	
Female	9	53	10	45	0.64
**Tobacco use**					
Yes	10	59	15	68	
No	7	41	7	32	0.55
**Alcohol use**					
Yes	5	29	7	32	
No	12	71	15	68	0.87
**Histology**					
Adenocarcinoma	17	100	22	100	1.00
**Tumor grade**					
Well differentiated	2	12	1	4	
Moderately differentiated	11	65	14	64	
Poorly differentiated	4	23	7	32	0.64
**T category**					
T1-T2	15	88	13	59	
T3-T4	2	12	9	41	0.05
**Nodal status**					
Negative (N0)	12	71	11	50	
Positive (N1, N2, N3)	5	29	11	50	0.20
**Distant metastasis**					
Yes	4	24	2	9	
No	13	76	20	91	0.22
**Tumor stage**					
Ia/Ib, IIa/IIb	13	76	12	55	
IIIa/IIIb, IV	4	24	10	45	0.16
**Outcome**					
Alive with disease	7	42	10	45	
Dead of disease	10	58	12	55	0.79

There are no statistically significant differences between clinical characteristics of patients in the training and validation sets.

### RNA extraction

Fresh-frozen tissues were subjected to frozen section analysis, performed by an expert lung pathologist (JD), in order to ensure the presence of >90% tumor or normal cells in samples collected by surgery. Fresh-frozen tissue samples were macrodissected, before RNA extraction, in order to isolate tumor or normal cells and samples were fragmented and lysed using the Precellys 24 lysing/homogenization system (Berting Technologies, Rockville, MD, USA) for 10s at 6,500 rpm. RNA extraction was performed using the miRNeasy Mini Kit (Qiagen, Hilden, Germany), following the manufacturer's protocol. Samples obtained from FFPE tissue blocks were needle microdissected using the stereo microscope Leica EZ4 (Leica Microsystems, Wetzlar, Germany) before RNA extraction, in order to isolate the target tumor or normal cell populations. RNA from FFPE samples was isolated using the *RecoverAll Total Nucleic Acid Isolation* kit (Ambion/Life Technologies, Carlsbad, CA, USA), following a previously reported protocol with modifications to improve RNA yield [45]. RNA samples were quantified using *NanoDrop 8000* (Thermo Fisher Scientific, Waltham, MA, USA) and quality was assessed using *Bionalyzer 2100* (Agilent Technologies, Santa Clara, CA, USA), following the manufacturer's protocol. RNA samples were stored at −80°C until use for library preparation.

### miRNA transcriptome sequencing (miRNA-Seq) and bioinformatic data analysis

RNA (1μg) from training set samples (*N* = 24) was used for library preparation, cluster generation and miRNA-Seq using the MiSeq system (Illumina, San Diego, CA, USA) at the Laboratory of Biotechnology, University of São Paulo (USP), Piracicaba, SP. Sequencing comprised *in vitro* cloning of RNA fragments in a solid platform. MiSeq platform generated 50bp single-read fragments. Briefly, library preparation used the *TruSeq Small RNA Sample Preparation* kit (Illumina, San Diego, CA, USA); 1μg RNA was used for adaptor ligation, which contains a ligation site for the sequencing primer, used to identify samples comprising an RNA pool and another ligation site for the flow cell primers, which are used for fragment amplification by PCR. cDNA libraries were obtained by PCR amplification following 11 cycles of 98°C for 30s, 98°C for 10s; 60°C for 30s; 72°C for 15s and 72°C for 10 min. Libraries were subjected to agarose gel electrophoresis for miRNA isolation; cDNA samples were then ethanol precipitated and quantified using Qubit 2.0 Fluorometer (Invitrogen/Life Technologies, Carlsbad, CA, USA). In the clustering step, fragments ligated to adaptors were denatured for double strand separation, allowing single strand molecules to bind primers in the flow cell and to produce multiple copies of specific fragments by solid phase PCR amplification. Transcriptome sequencing was performed using the MiSeq Reagent Kit v2 (50 cycles). All steps followed the manufacturer's instructions.

Data analysis included reads quality assessment using FastQC [46] and reads cleaning assessment by *CutAdapt* [47]. Reads alignment was performed based on hg19 reference genome (https://genome.ucsc.edu/cgi-bin/hgTracks?hgsid=12832096&chromInfoPage=) using *Bowtie1* [48] followed by *HT-Seq* [49] for annotation and quantification of aligned sequences. Data normalization [50] and miRNA differential expression analysis were performed using *edgeR* (Bioconductor/R) v.3.0 [51–53].

### Integrative bioinformatic analysis of published miRNA data sets

Our goal was to integrate our miRNA-Seq findings with miRNA expression changes in lung adenocarcinoma. We have summarized results of the 8 different studies comparing miRNA expression in lung adenocarcinoma and normal tissues [30, 37, 54–59] (Supplementary Table S2). Full text and (if applicable) Supplementary Data were carefully examined and miRNAs with significantly altered expression were extracted from each study. miRNA names were standardized according to the miRNA database miRBase (v.19) [60]. Based on data provided, all miRNAs were classified as either over- or under-expressed, and ranked according to reported statistical significance. Examining 8 studies we obtained 16 different rankings, 8 rankings for over- and 8 for under-expressed miRNAs. To identify consistently deregulated miRNAs, rankings were subjected to robust rank aggregation analysis implemented as R package RobustRankAggreg (v.1.1) [61]. This analysis detects miRNAs that are ranked consistently better than expected under null-model assuming that all studies are non-informative and input rankings thus contain only randomly ordered miRNAs. Using this analysis we assigned *p*-values as significance scores to each reported miRNA. The stability of resulting significance score was then assessed by the leave-one-out validation, in which the same analysis was repeated 8 times, each time excluding one of the rankings. Acquired *p*-values from each round were finally averaged into corrected *p*-value. Finally, miRNAs whose corrected *p*-value was less than 0.05 were further considered as consistently deregulated. Consistently reported miRNAs overlapping with those we identified herein, we referred to as “known”. miRNAs reported by at least one of the previous studies and overlapping with those we identified, we referred to as “reported”. miRNAs identified herein that were not reported by any of the studies are referred to as “novel”.

### Integrative bioinformatic analysis of published gene expression data sets

We have analyzed 10 publicly available gene expression datasets [30, 62–69] and GSE31547 (Supplementary Table S3), from studies on primary human lung adenocarcinoma and containing at least one histologically normal tissue sample for comparison. To enable uniform processing and analysis and to improve comparability of results, we chose only datasets produced using Affymetrix platforms. Each dataset was first separately normalized and summarized using Bioconductor project's package gcrma (GeneChip Robust Multiarray Averaging v.2.36.0) (http://watson.nci.nih.gov/bioc_mirror/packages/2.13/bioc/html/gcrma.html) [70]. For each individual dataset, we then evaluated differential gene expression using Bioconductor's limma package (v.3.18.13) [71]. Based on expression fold change, genes were classified as either over- or under-expressed, and then ranked according to statistical significance, which was evaluated by *q*-value (adjusted *p*-value). Analyzing 10 datasets, we obtained 20 unique rankings, 10 for over- and 10 for under-expressed genes. To identify consistently deregulated genes, obtained rankings were subjected to the same robust rank aggregation analysis as described for miRNA expression datasets, including leave-one-out cross-validation of the results. Genes with *p* < 0.05 were considered as consistently deregulated.

### miRNA-transcription factor (TF) regulatory network

To identify targets of differentially expressed miRNAs and relationships among them, we integrated data from multiple independent sources into miRNA-TF regulatory interactions. Knowledge of human TFs and their respective targets were obtained from four different databases, namely: ChEA (ChIP Enrichment Analysis) [72], ITFP (Integrated Transcription Factor Platform) [73], PAZAR [74], and TRED (Transcriptional Regulatory Element Database) [75]. These data were either downloaded as flat files (ITFP, PAZAR), manually collected (ITFP), or acquired from the web-based interactive application (ChEA). Additional data were obtained from TF:target pairs from human fetal lung [76]. Names of TFs and their respective targets as obtained from these databases were first standardized according to HGNC symbol checker (HUGO Gene Nomenclature Committee; http://www.genenames.org/cgi-bin/symbol_checker) and then concatenated into a single list comprising all the unique TF:target pairs. Those appearing in at least two sources were kept for further analysis, while the remaining ones were removed. We used mirDIP (microRNA Data Integration Portal, v.2.0; http://ophid.utoronto.ca/mirDIP) [77] to acquire list of targets of significantly deregulated miRNAs (*p < 0.005* and False Discovery Rate (FDR) < 0.01). In our search we considered only miRNA-target relationships among the top third of all predictions and from at least three different databases. Target gene names were standardized by HGNC symbol checker. As a result we obtained molecular interactions networks among differentially regulated miRNAs and their gene targets, either direct, or affected indirectly through their upstream TFs. Next, we integrated our data with previously published gene expression profiles to identify consistently deregulated genes in lung adenocarcinoma. For each deregulated miRNA, we evaluated statistical significance of enrichment of its downstream neighborhood of order 2 by deregulated genes (*p*-values calculated by hypergeometric test). Similarly, for each consistently deregulated gene, we have evaluated statistical significance of enrichment of its upstream neighborhood of order 2 by deregulated miRNAs. Order 2 neighborhoods were used rather than considering only the direct neighbors, since miRNA deregulation may affect the expression of its indirect targets through targeting transcription factors, while not involving alteration of the expression of a transcription factor itself. This is due to mechanisms of miRNA-mediated gene silencing that, depending on the target mRNA sequence, involves translational repression rather than mRNA degradation [40]. Data were visualized using NAViGaTOR 2.3.2 [78, 79]. Original miRNA-TF-gene regulatory network in NAViGaTOR 2 XML file format (http://ophid.utoronto.ca/navigator) is available at http://www.cs.utoronto.ca/~juris/data/Oncotarget16).

### Protein-protein interaction (PPI) network assembly and analysis

This analysis was performed to assemble PPI networks among gene targets of deregulated miRNAs. We used Interologous Interaction Database (I2D) (http://ophid.utoronto.ca/i2d, v.2.3) [80, 81], a database of protein-protein interactions for assembly of PPI networks among genes deregulated by differentially expressed miRNAs. Gene symbols were first converted to UNIPROT IDs by using Bioconductor's annotation package (Carlson M. *org.Hs.eg.db: Genome wide annotation for Human*. R package version 3.1.2; http://www.bioconductor.org/packages/release/data/annotation/html/org.Hs.eg.db.html). We then used NAViGaTOR v.2.3.2 [79] to assemble PPI networks comprising genes and their direct neighbors as nodes, and direct physical protein interactions as edges. To test significance of the interconnectedness between the nodes of the obtained PPI network, we generated 1e+5 random PPI networks. Each random PPI network was generated using a set of 148 seed genes (same as the number of genes found deregulated by the differentially expressed miRNAs) randomly chosen from the miRNA-TF regulatory network, by the same procedure as described above. For each random network, we then measured the number of direct PPI connections between the seed genes, as found in I2D. The resulting empirical distribution of the number of direct connections was used to derive the statistical significance of interconnectedness in the actual PPI network. The same random networks were similarly used to test significance of the enrichment of the actual PPI network by the prognostic genes. Resulting network was visualized using NAVIGaTOR 2.3.2 [78,79], and is provided in NAVIGaTOR 2 XML file format (http://ophid.utoronto.ca/navigator) available at http://www.cs.utoronto.ca/~juris/data/Oncotarget16).

### Validation of miRNA expression

Significantly deregulated miRNAs were validated using a TaqMan^®^ Array Human MicroRNA platform (Life Technologies, Foster City, CA, USA), as previously described [82]. We used the QuantStudio 12K system (Life Technologies, Foster City, CA, USA). Global data normalization was performed in Expression Suite software (Life Technologies, Foster City, CA, USA) and miRNA expression profiles were determined using RQ Manager v.1.2 software (Life Technologies, Foster City, CA, USA).

### Statistical analyses

Statistical analyses were performed to correlate deregulated miRNA expression with clinical and histopathological data of patients. Categorical variables were described using frequencies and percentages and continuous variables were summarized using mean and median (range) values. We used Mann-Whitney test and Fisher's exact test for comparisons between groups. The Kaplan-Meier method was used to estimate the curves from the observed survival times. The survival curves of any two groups were compared using the log rank test. Statistical analyses were performed by statistical software SAS version 9.3 for Windows (SAS Institute Inc., Cary, NC, USA). Statistically significant difference was defined as *p* < 0.05.

## SUPPLEMENTARY MATERIAL FIGURES AND TABLES


