# Supplementary material for: Integrative transcriptome analysis identifies deregulated microRNA-transcription factor networks in lung adenocarcinoma
Source: Oncotarget. 2016 Apr 12;7(20):28920–34. doi: 10.18632/oncotarget.8713 (PMC5045367; doi:10.18632/oncotarget.8713)
Supplement: Supplementary file 1 [file oncotarget-07-28920-s001.pdf]

Integrative transcriptome analysis identifies deregulated microRNA-transcription factor networks in lung adenocarcinoma

Supplementary Material

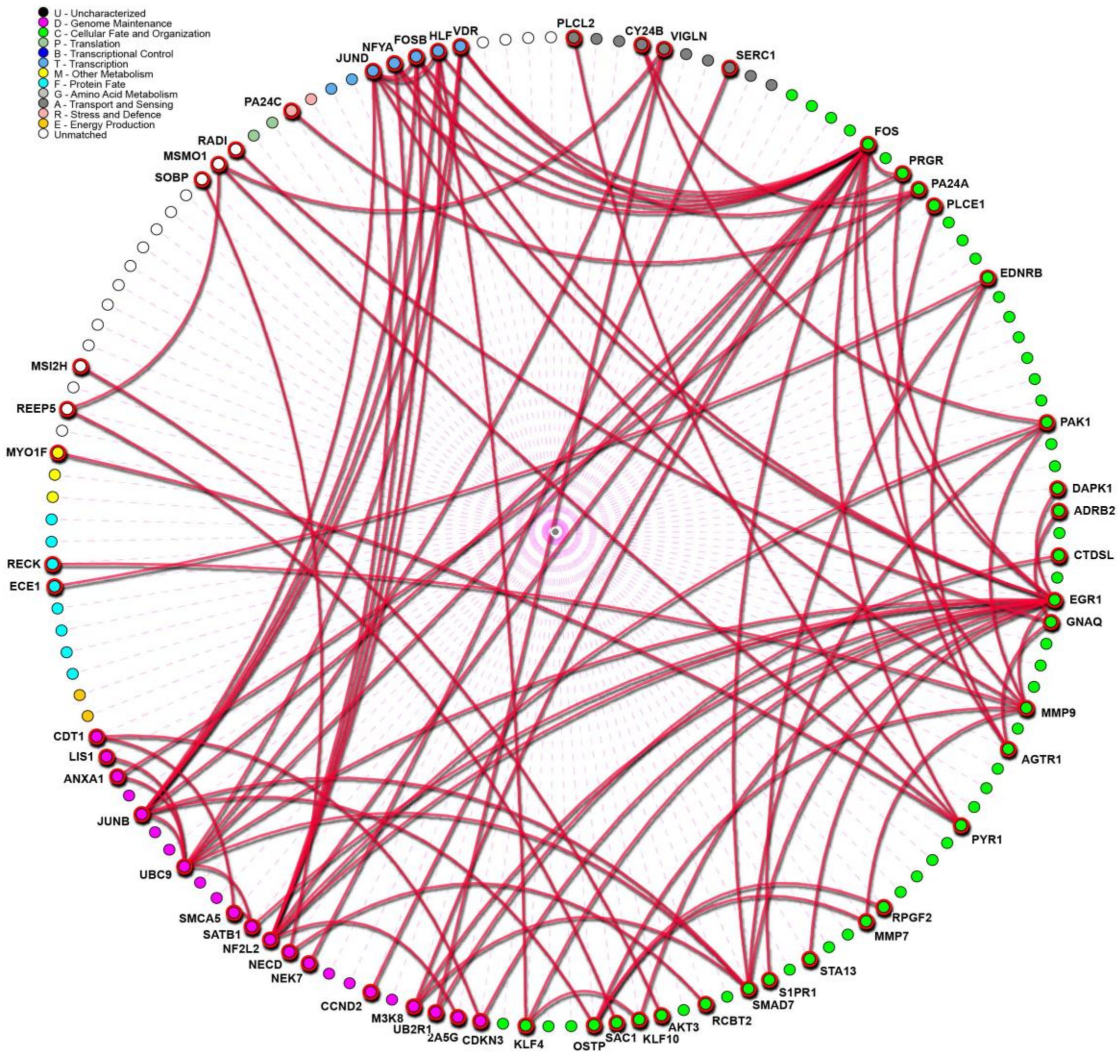

Supplementary Figure 1. PPI-network of commonly deregulated genes in lung adenocarcinoma. 148 genes (in circular layout), which have been consistently reported as deregulated in lung adenocarcinoma, and whose upstream neighborhood (order 2) is significantly enriched by differentially expressed miRNAs, were connected by PPIs ($p<0.001$; see PPI network analysis). 58 of these genes, which were directly connected ($p=0.044$) are labeled by their symbols. Network comprises 4,324 nodes in total, out of which 469 belong to lung cancer prognostic genes ($p=0.036$).

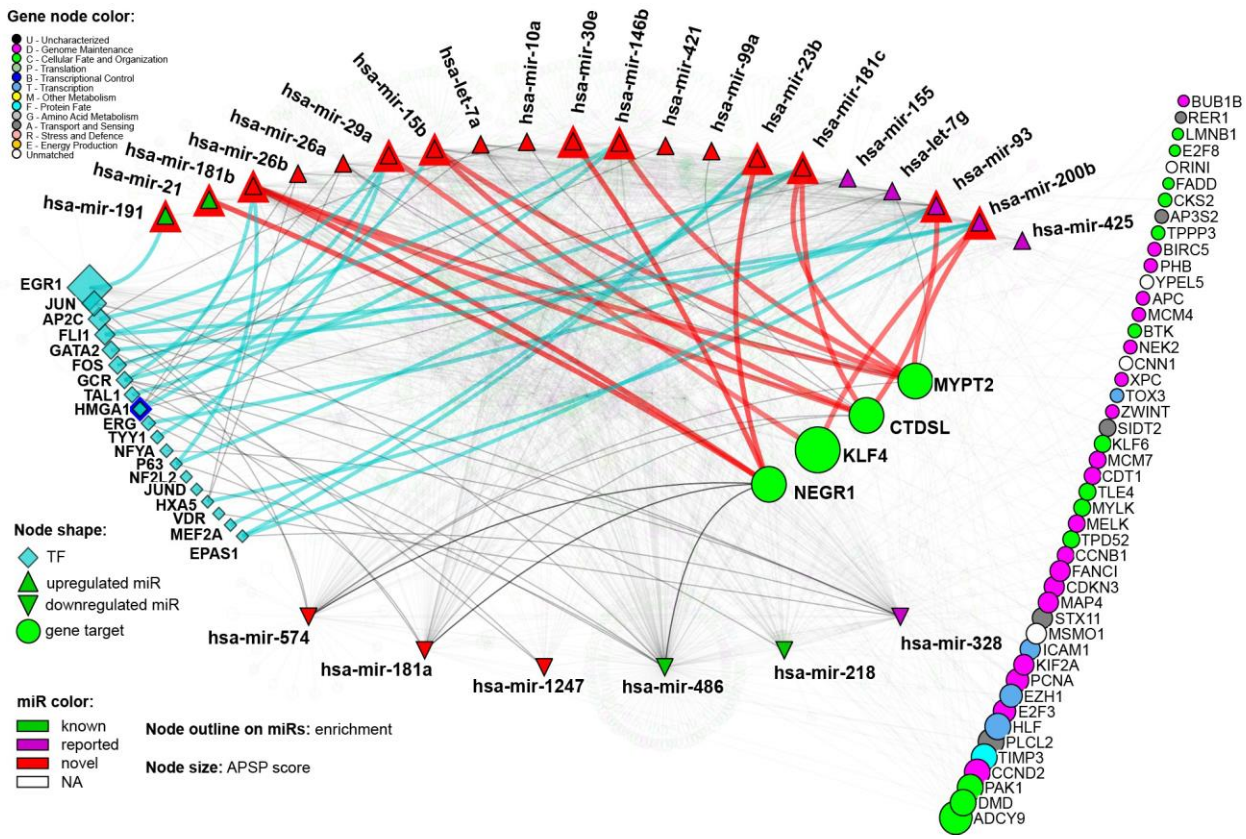

Supplementary Figure 2. miRNA-TF-gene regulatory network. Differentially expressed miRNAs whose downstream neighborhood is significantly enriched in deregulated genes (*p<0.05*), are highlighted. Among these, miRNAs miR-15b, miR-23b, miR-29a, miR-30e, miR-146b, miR-181b and miR-181c, have not been previously reported in lung adenocarcinoma compared to normal lung tissues from same patients (red color triangles). There are 705 genes that have been consistently reported as deregulated in tumors and whose upstream neighborhood is significantly enriched in differentially expressed miRNAs (p<0.05). 48 of these belong to lung cancer prognostic genes, ordered using an APSP score that measures centrality by calculating shortest paths among all nodes (right side). There are also 19 TFs whose upstream neighborhood is enriched by differentially expressed miRNAs (p<0.05); (list ordered based on APSP score on the left side), among which *HMGA1* (highlighted in blue) is a lung cancer prognostic gene.

Supplemental Table 1: List of 148 deregulated genes in lung adenocarcinoma.

Official gene symbol (hgnc_symbol)	uniprot_swissprot ID
KLF4	O43474
EGR1	P18146
FOS	P01100
PPP1R12B	O60237
NEGR1	Q7Z3B1
SLC2A3	P11169
PHACTR2	O75167
PNRC2	Q9NPJ4
PALLD	Q8WX93
ADCY9	O60503
RTKN2	Q8IZC4
PTPRD	P23468
CTDSPL	O15194
TFRC	P02786
EDNRB	P24530
FMO2	Q99518
CBFA2T3	O75081
PPP2R5C	Q13362
DIXDC1	Q155Q3
PLCL2	Q9UPR0
KLF10	Q13118
AKT3	Q9Y243
KLF9	Q13886
PAK1	Q13153
DMD	P11532
HLF	Q16534
CCND2	P30279
MTMR9	Q96QG7
STARD9	Q9P2P6
SLC1A1	P43005
SMAD7	O15105
AGK	Q53H12
FOSB	P53539
TBX5	Q99593
MRPS23	Q9Y3D9
SAMHD1	Q9Y3Z3
NEK7	Q8TDX7
CCDC141	Q6ZP82
FRMD4B	Q9Y2L6
WIF1	Q9Y5W5
KIAA1462	Q9P266
DOCK4	Q8N1I0
WASF3	Q9UPY6
EPB41L3	Q9Y2J2

RAPGEF2	Q9Y4G8
PCDH17	O14917
TRAK2	O60296
OSBPL2	Q9H1P3
CHL1	O00533
METAP1	P53582
HDLBP	Q00341
STEAP3	Q658P3
ARHGEF3	Q9NR81
KL	Q9UEF7
RMI2	Q96E14
PAFAH1B1	P43034
S1PR1	P21453
STARD13	Q9Y3M8
SVEP1	Q4LDE5
DLC1	Q96QB1
MMP9	P14780
MTX2	O75431
PLA2G4A	P47712
RCBTB2	O95199
B3GNT1	O43505
ACYP1	P07311
SERINC1	Q9NRX5
SLC7A5	Q01650
SESN1	Q9Y6P5
FAM83A	Q86UY5
DAPK1	P53355
CDC34	P49427
MSMO1	Q15800
SSFA2	P28290
KLRF1	Q9NZS2
NTS	P30990
GYG2	O15488
AGTR1	P30556
KDELR3	O43731
RPGR	Q92834
KIF2A	O00139
HGF	P14210
GREM1	O60565
ADRB2	P07550
PLCE1	Q9P212
RECK	O95980
RDX	P35241
MFAP3L	O75121
ZNF423	Q2M1K9
NFE2L2	Q16236
CDKN3	Q16667
JUND	P17535
ABI3BP	Q7Z7G0
OSBPL11	Q9BXB4

SOBP	A7XYQ1
OTUD1	Q5VV17
DECR2	Q9NUI1
SAPCD2	Q86UD0
SYT12	Q8IV01
HOXA5	P20719
MARC2	Q969Z3
JUNB	P17275
NFYA	P23511
MYO1F	O00160
UBE2I	P63279
MMP7	P09237
SPP1	P10451
ADAM28	Q9UKQ2
ECE1	P42892
CDT1	Q9H211
LZTFL1	Q9NQ48
GALNT2	Q10471
ACKR4	Q9NPB9
N4BP2L1	Q5TBK1
HMBS	P08397
MAP3K8	P41279
GLRX	P35754
COASY	Q13057
REEP5	Q00765
PGR	P06401
USP12	O75317
CYBB	P04839
SACM1L	Q9NTJ5
SATB1	Q01826
MSI2	Q96DH6
CAD	P27708
TMED3	Q9Y3Q3
CLDN4	O14493
CLCN7	P51798
ANXA1	P04083
GNG11	P61952
HSD17B6	O14756
FNBP1	Q96RU3
METTL21B	Q96AZ1
DOCK6	Q96HP0
SMG5	Q9UPR3
PALMD	Q9NP74
AIFM1	O95831
PLA2G4C	Q9UP65
ST6GALNAC5	Q9BVH7
SMARCA5	O60264
VDR	P11473
GNAQ	P50148
SLC16A3	O15427

NDN	Q99608
HHIP	Q96QV1
MIS18A	Q9NYP9
SLC52A2	Q9HAB3

Supplemental Table 2: Summary of miRNA datasets in lung adenocarcinoma.

Histology	Number of samples (tumor/normal)	Author
**ADC, SCC**	104/104	Yanaihara et al. 2006
**ADC**	20/10	Cho et al. 2009
**ADC**	20/8	Crawford et al. 2009
**ADC**	112/56	Jang et al. 2012
**ADC**	40/20	Yu et al. 2010
**ADC**	12/6	Dacic et al. 2010
**ADC, SCC**	12/6	Lee et al. 2011
**ADC, SCC**	216/108	Ma et al. 2014

Note, if multiple histologies were studied, only miRNAs clearly stated as differentially expressed in adenocarcinoma as compared to normal tissue, were taken into consideration.
Reference citations in supplemental methods file.

Supplemental Table 3: Summary of gene expression datasets in lung adenocarcinoma.

**Platform**	**Histology**	**Number of samples**	**Author**
Hu6800	ADC	96/10	Beer et al. 2002
HG-U95Av2	ADC	228/17	Bhattacharjee et al. 2001
HG-U133p2	ADC, SCC, LCC	156/64	Hou et al. 2010
HG-U133a	ADC	107/50	Landi et al. 2008
HG-U133p2	ADC	246/20	Okayama et al. 2012
HG-Focus	ADC, SCC	25/5	Rohrbeck et al. 2008
HG-U95Av2	ADC	39/19	Stearman et al. 2005
HG-U133a	ADC	54/27	Su et al. 2007
HG-U133a	ADC	50/20	*Girard et al. Unpublished data GEO: GSE31547
HG-U133a	ADC	58/9	Yu et al. 2005

Note, datasets containing multiple cancer types were reduced to adenocarcinoma samples and paired normal tissue samples only. Samples from other cancer types were excluded.

*Data from Girard et al. were obtained from Gene Expression Omnibus Database (GEO) under given accession number.
